# Assessment of the therapeutic potential of lutein and beta-carotene nanodispersions in a rat model of fibromyalgia

**DOI:** 10.1038/s41598-023-46980-6

**Published:** 2023-11-12

**Authors:** Nourhan S. Elkholy, Haitham S. Mohammed, Medhat W. Shafaa

**Affiliations:** 1 Medical Biophysics Division, Physics Department, Faculty of Science, Helwan University, Cairo, Egypt; 2 Nawah Scientific Co., Cairo, Egypt; 3 Biophysics Department, Faculty of Science, Cairo University, Giza, Egypt

**Keywords:** Biochemistry, Biophysics, Neuroscience, Diseases, Nanoscience and technology

## Abstract

Fibromyalgia (FM) is a chronic disorder characterized by widespread musculoskeletal pain, fatigue, and cognitive impairment. Despite the availability of various treatment options, FM remains a challenging condition to manage. In the present study, we investigated the efficacy of formulated nanodispersions of lutein and beta-carotene in treating FM-related symptoms induced by reserpine in female Wistar rats. Several techniques have been implemented to assess this efficacy at various levels, including biochemical, bioelectrical, and behavioral. Namely, oxidative stress markers, monoamine levels, electrocorticography, pain threshold test, and open field test were conducted on control, FM-induced, and FM-treated groups of animals. Our results provided compelling evidence for the efficacy of carotenoid nanodispersions in treating FM-related symptoms. Specifically, we found that the dual action of the nanodispersion, as both antioxidant and antidepressant, accounted for their beneficial effects in treating FM. With further investigation, nano-carotenoids and particularly nano-lutein could potentially become an effective alternative treatment for patients with FM who do not respond to current treatment options.

## Introduction

Fibromyalgia (FM) is a chronic condition characterized by widespread pain throughout the body, often accompanied by fatigue, sleep disturbances, cognitive impairment, and other comorbidities such as depression^1–4^. FM studies in Europe and South America reported that 3.3–8.3% of the population are FM patients and its severity increases with aging^5^. Family history and having rheumatic disease increase the probability of FM occurring. 40% of patients who ask for pain assessment in pain clinics have the criteria of FM^5^.

It is a complex and multifaceted disease that involves a variety of factors. Central sensitization and chronic oxidative stress are two fundamental mechanisms that have been identified as playing key roles in the development and maintenance of FM^6,7^. Central sensitization refers to a phenomenon in which the central nervous system becomes hypersensitive to pain signals, leading to the perception of pain even without a noxious stimulus^8,9^. In FM, central sensitization is thought to be caused by changes in the function of pain-processing pathways in the central nervous system, including alterations in neurotransmitter function and abnormal sensory processing^10,11^. Chronic oxidative stress, on the other hand, is a condition where there is an imbalance between the production of reactive oxygen species (ROS) and the ability of the body to detoxify and repair the damage caused by these molecules^12,13^.

Both central sensitization and chronic oxidative stress are complex processes that involve multiple pathways and mechanisms, and the exact relationship between these factors and the development of FM is not well understood. However, research suggests that interventions that target these processes, such as medications that modulate neurotransmitter function or antioxidants that reduce oxidative stress, may be effective in reducing pain and other symptoms in people with FM^14–16^.

Some evidence suggests that abnormalities in monoamine function may be involved in the development of FM^17,18^. For example, serotonin (5-HT) is a monoamine involved in regulating mood, sleep, and pain perception, among other functions^19^. Some studies have found that people with FM have lower levels of 5-HT in their cerebrospinal fluid than healthy controls, which may contribute to the development of pain and other symptoms^19^. Similarly, norepinephrine (NE) and dopamine (D) are monoamines that participate in pain modulation and mood regulation. Some studies have suggested that abnormalities in the function of these neurotransmitters may be involved in the development of FM^20,21^. In addition to these monoamines, other neurotransmitters, and neuropeptides have also been implicated in the development and maintenance of FM, including substance P, glutamate, and GABA^22^.

FM is more prevalent, in severity score, and number of tender points in females than in males, with estimates suggesting that women are two to three times more likely to be diagnosed with FM than men, the most common symptom in women is musculoskeletal pain^5,23,24^. The reasons for this gender disparity are not yet fully understood, but several factors have been proposed to contribute to this phenomenon^23^. One possible explanation is hormonal differences between males and females. Female sex hormones, such as estrogen and progesterone, have been shown to modulate pain perception and may play a role in the development of FM^25,26^. Women also experience hormonal fluctuations throughout the menstrual cycle, pregnancy, and menopause, which may contribute to the development or exacerbation of FM symptoms^27^. Another possible explanation is differences in pain processing between males and females. Studies have shown that females have a lower pain threshold and greater sensitivity to pain than males, which may contribute to the development and maintenance of FM^28^. Psychosocial factors, such as stress, trauma, and social support, have also been implicated in the development of FM and may contribute to the gender disparity in FM^29^. Women may be more likely to experience stress and trauma, and may also have different coping strategies and social support networks than men, which may contribute to the development and maintenance of FM^30^.

Animal models of FM are used to study the underlying pathophysiology of the condition and to assess potential treatments. However, it is important to note that no animal model can fully mimic the complex and multifactorial nature of FM in humans^31^. One commonly used animal model of FM is the reserpine-induced myalgia model^32^. Reserpine is a drug that depletes the levels of monoamine neurotransmitters in the nervous system^33^. In this model, rats are treated with reserpine, which leads to the development of muscle hypersensitivity and other symptoms that resemble FM in humans. This model has been used to study the role of monoamine neurotransmitters in the development of FM and to evaluate potential treatments that target these neurotransmitters mainly by inhibiting the activity of vesicular monoamine transporter (VMAT) and increasing the oxidative stress in nerve cells^32,34,35^.

The intramuscular acidic saline injection model is another commonly used model that uses repeated injections of acidic saline into the gastrocnemius muscle to develop widespread pain^34,35^. Another animal model of FM is the intermittent cold stress model as rats are exposed to repeated sudden changes from room temperature to a cold environment to develop several abnormal physiologies^34,35^. The sound stress model used the same concept of stress by using repeated unpredictable sounds with a different range of amplitudes and frequencies with reported weaknesses^34,35^. Another animal model of FM is the repeated immobilization stress model. In this model, rats are repeatedly immobilized, which leads to the development of widespread pain. This model has been used to study the role of stress and other psychosocial factors in FM^36^. Other animal models of FM include the chronic constriction injury model, which involves the compression of a nerve to induce neuropathic pain^37^, and the ovariectomy model, which involves the removal of the ovaries to induce hormonal changes that may be associated with FM^37^.

Carotenoids are well-known as potent natural antioxidants^38,39^. Recently, carotenoids were found to be anti-inflammatory and antidepressant^40^. On the other side, carotenoids control the cell cycle^38,39,41^, cell-to-cell communication^38,39,41^, cell proliferation^38,41^, and cell transportation^38,39^ which nominates them to be a suitable therapeutic solution for fibromyalgia. The main classes of carotenoids are carotenes (as beta-carotene, alpha-carotene, and lycopene) and xanthophylls (as lutein, zeaxanthin, and canthaxanthin)^38,41^. Lutein and beta-carotene are the most widespread carotenoids^42^. The major difference in their chemical structures is the -OH group of lutein which diverges their distribution in the cell membrane, solubility, stability, and activity^38,41^. Although they can be found in fruits, vegetables, marines, fungi, and bacteria, carotenoids in these nutrition sources degrade because of the acidic pH of the stomach, and with low concentrations reaching the blood, the activity is minimal^40^. On the other side, the hydrophobicity of carotenoids limits their pharmaceutical uses, they need a suitable nanosystem to reach their target with higher activity^41,42^. The therapeutical use of carotenoids is suggested to be separate because they can affect the absorption and activity of each other^43^.

The nanodispersions prepared using surfactants like polysorbate 80 demonstrated promising in vivo outcomes, making them a commonly employed approach in the treatment of neurological disorders. Previous studies have demonstrated that brain capillary endothelial cells in tissue cultures can absorb nanoparticles coated with surfactants, such as polysorbate 80/ Tween 80 (T80). Specifically, nanoparticles coated with polysorbate 80 were found to have a significant effect. Additionally, the use of 1% nonionic surfactant T80 was shown to reduce the accumulation of certain drugs in the liver and spleen^44^. The critical concentration of T80-coated nanoparticles for optimal blood translocation was found to be between 1–2% w/w or w/v^45^. However, increasing the dosage of T80 to 3–30 mg/kg was reported to disrupt the blood–brain barrier (BBB) in mice^46^. Molecular docking studies have revealed that T80 interacts with apolipoprotein E through π−π interactions and hydrogen bonding, which may be the mechanism for its transmembrane permeation across the BBB^47^.

Based on our previous in-vitro research^48^, we have hypothesized that the utilization of nano-dispersed lutein (Nano-Lut) and beta-carotene (Nano-Bc) could serve as a promising and safe therapeutic approach for FM. Nevertheless, to validate this hypothesis, it is essential to investigate the effects of these substances in an in vivo setting.

The objective of this study is to investigate the potential therapeutic efficacy of Nano-Lut and Nano-Bc on the reserpine-induced FM model in female Wistar rats. Various parameters were assessed, including measuring oxidative stress markers and monoamine neurotransmitter levels, analyzing electrocorticography (ECoG) data, and conducting behavioral tests. By examining these parameters, we aim to better understand the potential therapeutic benefits of nano-carotenoids in treating FM.

## Materials and methods

### Drugs and chemicals

Reserpine of 99% purity (HPLC), Sod. Pentobarbital, carotenoids reference standards, and synthetic beta-carotene (97%) were provided by Sigma (St. Louis, Mo, USA). The solutions were made in ultra-pure deionized water. Marigold flowers were purchased in Egypt at local markets. Lutein was extracted from Marigold flowers (*Tagetes erecta* L.), which were collected from a local place according to the procedures that comply with institutional guidelines and legislation. The purity of lutein extracted was determined to be 90% using a spectrophotometer, HPLC, TLC-mass, and Proton and Carbon NMR, as previously described^48^. The solutions were created using distilled ultra-pure water. All other reagents and solvents used in this work were of HPLC grade.

### Experimental animals

Thirty-two adult female Wistar rats weighing 200 ± 30 g were used in this study. The rats were housed in plastic cages (5–6 animals per cage) under controlled conditions of temperature (25 ± 2 °C) and light cycle (12 h light, 12 h dark), with standard food pellets and tap water provided ad libitum. Following a one-week acclimatization phase, experimental procedures were conducted in accordance with international animal care and ethical guidelines, approved by Cairo University’s local committee on animal care and use (CU/I/F/28/19), and reported in accordance with ARRIVE guidelines.

### Experimental design

Animals were divided into 4 groups: control group, FM model group, Nano-Bc, and Nano-Lut treatment groups. Animals were housed in cages with dimensions of 90 × 60 × 50 cm, accommodating 5–6 rats per cage. The control group received the drug’s vehicle (0.5% acetic acid with distilled water and injected subcutaneously in a volume of 1 mL/kg) and intravenous (IV) physiological saline in the tail for three days and continued to receive daily injections at noon for two weeks, starting on the fifth day. For the FM model, the animals were administered three consecutive days of subcutaneous (SC) injections of reserpine (1 mg/kg), which was dissolved in glacial acetic acid and then diluted with double-distilled water to a final concentration of 0.5% acetic acid, the dose adjusted to be injected in a volume of 1 mL/kg^49^. Following the three-day reserpine injections, the animals received daily injections of IV physiological saline in the tail at noon for two weeks, starting on the fifth day. The third and fourth groups received three days of SC reserpine injections, followed by daily injections at noon for two weeks starting on the fifth day of IV Nano-Bc (0.5 mg/kg) and Nano-Lut (0.5 mg/kg) treatment in the tail, respectively. Every three days, nanodispersions were freshly prepared and stored at 4 °C. Dosage was selected according to a pilot study (Data not shown). All tests were carried out on the animals after 1 h of the last IV injection.

### Preparation and characterization of carotenoid nanodispersions

Nano-Bc and Nano-Lut were prepared and characterized following our previously published protocol^48^. In brief, the solvent displacement method was used to create Nano-Lut and Nano-Bc. Nano-Lut was composed of 0.1% (w/w%) Tween 80 (T80) and 0.1% (w/w%) Lut, while Nano-Bc was composed of 1% T80 and 0.2% Bc to enhance absorption by brain capillary endothelial cells. The effective weight of Lut or Bc was determined using a spectrophotometer before preparing the nanodispersions. Additionally, Fourier-transform infrared spectroscopy (FTIR), transmission electron microscopy (TEM), and stability studies were conducted, in the present study, to evaluate the prepared nanodispersions.

#### Nanodispersion morphology by TEM

The size and morphology of Nano-Lut or Nano-Bc were examined using a negative stain transmission electron microscope HR-TEM (Tecnai, G20, FEI, Netherlands) operating at 200 kV. As a negative staining agent, an aqueous solution of phosphotungstic acid stain (2% w/v) was used. After staining, a 2-min wait at room temperature was followed by the removal of excess solution with filter paper and examination under the electron microscope. Each nanodispersion aliquot was applied to a transmission electron microscopy (TEM) grid (carbon-coated copper grid) in the amount of 20 μL. After 1 min, the excess solution was removed from the grid using filter paper^50^.

#### FTIR spectroscopy

Using Jasco FTIR-4100 spectrometer (Tokyo, Japan), FTIR spectra of lyophilized samples of Lutein (Lut), Beta-carotene (Bc), T80, Nano-Lut, and Nano-Bc deposited in KBr discs were scanned. Scanning was performed at room temperature in the 4000–400 cm^−1^ range at a speed of 2 mm/s and a resolution of 4 cm^−1^. The data were normalized and expressed as means values.

#### Stability study

To assess the stability of the prepared nanodispersions, the mean size, polydispersity index (PDI), zeta potential (ZP), and drug content were measured at various time points. Specifically, measurements were taken at 0, 1, 2, 3, 4, 6, and 8 weeks after preparation at 4 °C, as well as at 30 °C and 40 °C for 0, 1, 2, 3, and 4 weeks. To eliminate the potential impact of light exposure, three independent preparations of Nano-Lut and Nano-Bc were assessed fresh (at the zero-time interval) and then stored in opaque glass vials. At each interval, 2 mL of each vial was collected and used for the measurements.

#### Dynamic light scattering (DLS) measurement

The mean particle size and PDI of the prepared nanodispersions (diluted by a factor of 10) were determined using DLS with a particle sizing system (Zetasizer Nano ZS, Malvern, UK) at 25 °C. Each measurement was based on the intensity-based mean diameter calculated from an average of 12 runs done in triplicate. The results were expressed as an average of three independent measurements.

#### Zeta potential measurements

The zeta potential of the prepared samples (X10 dilution) was determined after dilution in ultra-pure deionized water at 25 °C using Zetasizer Nano ZS, Malvern, UK. The results were expressed as the average of three individual measurements.

#### Drug content measurement

The drug content was determined using the extraction method described by Tan et al.^51^, with some modifications. Aliquots of 1 ml of the nanodispersion and 3 mL dichloromethane (DCM) were vortexed vigorously for 2 min at room temperature to obtain the free amount of carotenoid in dispersion. The mixed sample was centrifuged at 5000 rpm for 5 min to collect the centrifuged sample’s DCM layer. The preceding procedure was repeated twice. Finally, the amount of DCM collected was combined in a tube and additional DCM was added to reach the absorbance acceptable range of 0.1–0.8. With DCM as the blank, the free amount of carotenoid was measured spectrophotometrically at 460 and 452 nm for Bc and Lut, respectively. Bc or Lut drug content in nanodispersions was calculated as the ratio between the recovered carotenoid amount by extraction to the initial amount of the freshly prepared nanodispersion at zero point.

### Neurochemical parameters determination

Calorimetric assay kits (Biodiagnostic Co., Egypt) were used to measure malondialdehyde (MDA), hydrogen peroxide (H_2_O_2_), nitric oxide (NO), and glutathione reduced (GSH) in brain cortical tissue from all groups. Fluorimetry (spectrofluorometer model Jasco-FP-6500, Japan) was used to determine the levels of monoamines (NE, DA, and 5-HT) in cortical tissue. The rats were decapitated after the treatment period, and their brains were immediately extracted and placed on an ice plate for dissection of the cortex. The cortex tissue in all animals was divided into 2 halves: the left half was used to determine monoamine levels, while the right half was used to measure oxidative stress. The tissue samples were kept at − 30 °C until they were measured. To measure the biochemical parameters, the tissues were perfused with a phosphate-buffered saline (PBS) solution (pH 7.4) containing 0.16 mg/ml heparin at the time of measurement. After homogenizing the tissues in a cold buffer (using 20% homogenate), they were centrifuged at 4000 rpm for 15 min at 4 °C. The supernatant that resulted was collected and stored at − 80 °C until further analysis^52^.

#### MDA determination

A 0.2 ml sample or standard was mixed well with 1 mL of 25 mM/L chromogen (thiobarbituric acid), covered, and heated in a boiling water bath for 30 min. 1 mL of chromogen was heated and cooled before adding 0.2 mL of the sample as a blank. At 534 nm, samples and standards were read against the blank^53,54^ (Deionized water was used as the blank). The following formula was used:

$$ {\text{Malondialdehyde}}\left[ {{\text{nM}}/{\text{g tissue}}} \right] = \frac{A\,\, of\,\, the\,\, sample}{{A\,\, of\,\, the\,\, standard}} \times \frac{10}{{g \,\,tissue\,\, used}} $$

#### H_2_O_2_ determination

For H_2_O_2_ determination in tissue, the Fossati et al.^55^ and Aebi^56^ method was used. In the presence of peroxidase, H_2_O_2_ reacts with 3,5-dichloro-2-hydroxybenzensulfonic (DHBS) acid and 4-aminophenazone (AAP) to form a quinonimine dye. 50 μL of 100 mM/L 3,5 dichloro-2-hydroxy benzene sulfonate in phosphate buffer (pH 7) was used as a chromogen, and 500 μL of 4-amino antipyrine peroxidase preservatives were added to 50 μL of the sample or standard, and 500 μL of chromogen and 500 μL deionized water were added to 50 μL of the blank sample. After incubating the mixtures for 10 min at 37 °C, the samples and standard were spectrophotometrically measured against a blank at 510 nm. The following formula was used:

$$ {\text{H}}_{{2}} {\text{O}}_{{2}} \left[ {{\text{mM}}/{\text{g\,\, tissue}}} \right] = \frac{A\,\, of\,\, the\,\, sample}{{A\,\, of\,\,the\,\,standard}} \times \frac{0.5}{{g\,\,tissue\,\,used}} $$

#### NO determination

Nitrite forms nitrous acid diazotizes sulfanilamide in acidic pH, and the product becomes azo dye when coupled with N(1-naphthyl) ethylenediamine (NEDA). 1 mL of sulfanilamide (10 mM/L) was added to 100 μL of sample, standard, or blank and mixed thoroughly before standing for 5 min before adding 100 μL of 1 mM/L NEDA to the samples and standard, mixed thoroughly, and stood for another 5 min. At 540 nm, spectrophotometry was used to compare samples and standards to their blanks^57^. The following formula was used:

$$ {\text{NO}}\left[ {\mu {\text{M}}/{\text{g tissue}}} \right] = \frac{A\,\,of\,\,the\,\,sample}{{A\,\,of\,\,the\,\,standard}} \times \frac{50}{{g\,\,tissue\,\,used}} $$

### GSH determination

Glutathione (GSH) reduces 5,5′ -dithiobis-(2-nitrobenzoic acid) (DTNB) and produces chromogen in direct proportion to GSH concentration^58^. 0.5 mL sample or the same amount of distilled water (blank) was mixed with 0.5 mL of 500 mM/L C_2_HCl_3_O_2_ acid. After 5 min at room temperature, the solution was subjected to centrifugation at 3000 rpm for 15 min. 0.5 mL of supernatant was added to 1 ml of buffer and 0.1 mL of 1 mM/L DTNB, mixed well, and left for 10 min before reading at 405 nm. The following formula was used:

$$ {\text{GSH}}\,{\text{amount }}\left[ {{\text{mM}}/{\text{g tissue}}} \right] = A\,\,of\,\,the\,\,sample \times \frac{2.22}{{g\,\,tissue\,\,used}} $$

#### Monoamines level determination

According to Ciarlone^59^, weighted samples of cortical tissue were added to cold acidified butanol (3 mL) in a small beaker and homogenized for 30 s before being placed in plastic tubes and centrifuged at 5000 rpm for 5 min. 2.5 mL of the supernatant was mixed with 1.6 mL of (0.2 N) acetic acid and 5 mL heptane before being vortexed for 30 s and centrifuged at 5000 rpm for 5 min. A spectrofluorometer with a 150 W xenon arc lamp source was used to measure the fluorescence of DA, NE, and 5-HT at various excitation and emission wavelengths. Excitation wavelengths were 320 nm, 380 nm, and 355 nm, while emission wavelengths were 370 nm, 460 nm, and 470 nm for DA, NE, and 5-HT, respectively.

### ECoG signals recording and analysis

ECoG signals recordings were performed on animals that were awake and moving freely in their experimental cage. Electrodes were implanted in the animal’s head 2 weeks before ECoG signals acquisition. The surgery was executed under anesthetic with sodium pentobarbital (40 mg/kg, i.p.). The animals were placed in the stereotaxic device (David Kopf Instruments, Tujunga, CA, USA) and holes were drilled in the frontal, parietal, and occipital regions of the skull at the following locations: ± 2 mm from Bregma, 2 mm lateral to the midline (ipsilateral), and 2 mm posterior to Lambda (reference electrode). The electrodes were 1 mm stainless steel screws. Dental cement (zinc polycarboxylate, Spofa-Dental-Praha, Czech Republic) was used to secure the electrodes in place, cover the skull, and act as a skin adhesive. Two skin stitches were applied to help the skin heal. Before the experiments began, a topical antibiotic was applied to the animal's head, and it was given two weeks for the animal to recover.

After the 2 weeks of the treatment, animals were then moved individually to a glass cage surrounded by a Faraday cage for sound attenuation and electrical isolation during ECoG signal recording. The animal was left in the cage for 5 min (adaptation period) before being connected to the ECoG recording setup via a commutator that allows its movement during signal recording. Signals were amplified, fed to a computer via ADC (National Instruments, USA), and displayed online on the computer's screen using custom-built software. To avoid the circadian variations of the ECoG signals, all recordings were performed at the same time of the day. Fast Fourier Transformation (FFT) and power spectrum construction were used to analyze the recorded ECoG signals. To obtain a quantitative analysis of the ECoG signals, power spectra were divided into five frequency bands: delta (1–4 Hz), theta (4.1–7 Hz), alpha (8–13 Hz), beta-1 (13.1–20 Hz), and beta-2 (20.1–35 Hz). Absolute and relative band powers were obtained and used for signal evaluation^52^.

### Pain threshold test

The hot plate test uses heat to determine the pain threshold. Individual rats were placed on a metal hot plate surface, and the temperature gradually increased from 40 to 50 °C within 30 s. The rat was placed in a large glass box on the hot plate so it couldn't escape, and the response latency was recorded. The time it took to observe a jumping response was considered the response latency. To avoid tissue damage, the animal was removed from the hot plate after 1 min if no response behavior was observed. The test was done at the end of the experiment (after the 2 weeks of the treatment) and repeated three times for each rat, and the average value was computed.

### Open field test (OFT)

The OFT can provide quantitative measurements of animal locomotory and exploratory behavior. During the test, the animal is placed in the center of a 40 × 50 × 50 cm plastic box. Under the box's transparent floor, 12 pieces of equal squared paper were placed, and the test was done after the treatment period. Several parameters are recorded for each animal for 10 min including the number of squares crossed, number of rearing, number of grooming, number of sniffing, and mobile and immobile time spent in the central and peripheral regions of the box. For each animal group, these parameters were calculated and averaged.

### Statistics

The mean value and standard deviation were used to express the results. A significant difference between groups was determined using one-way ANOVA followed by the post hoc Tukey test, with a P-value less than or equal to 0.05 considered significant for all statistical analyses.

## Results

### Nanodispersion morphology by TEM

**Figure 1:**
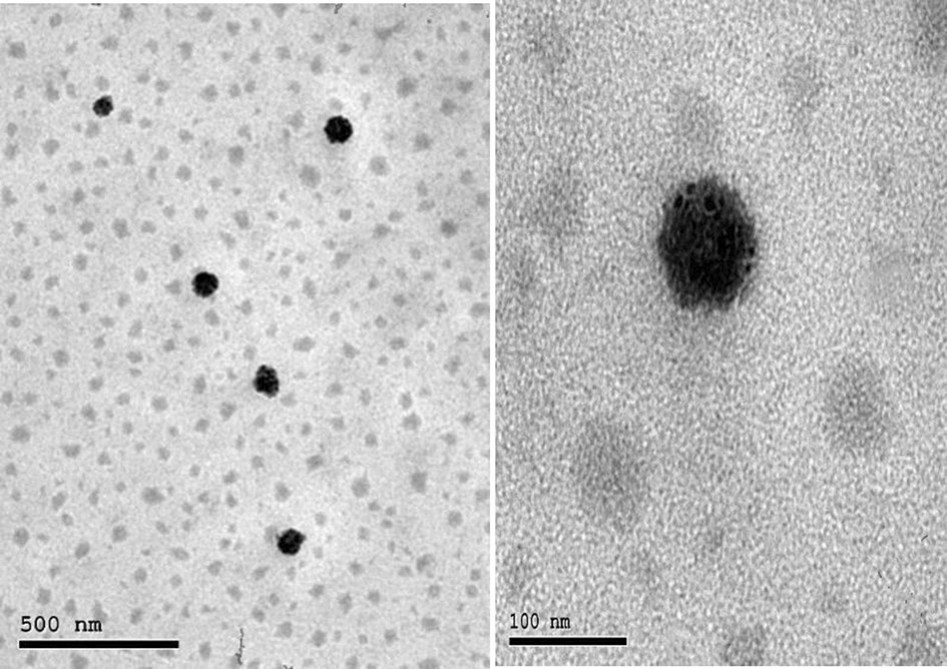
TEM images of lutein nanodispersion using solvent displacement method at a volume ratio of 1:9 (water phase: organic phase) using tween 80 showing the morphology at low magnification in the left and at high magnification in the right.

**Figure 2:**
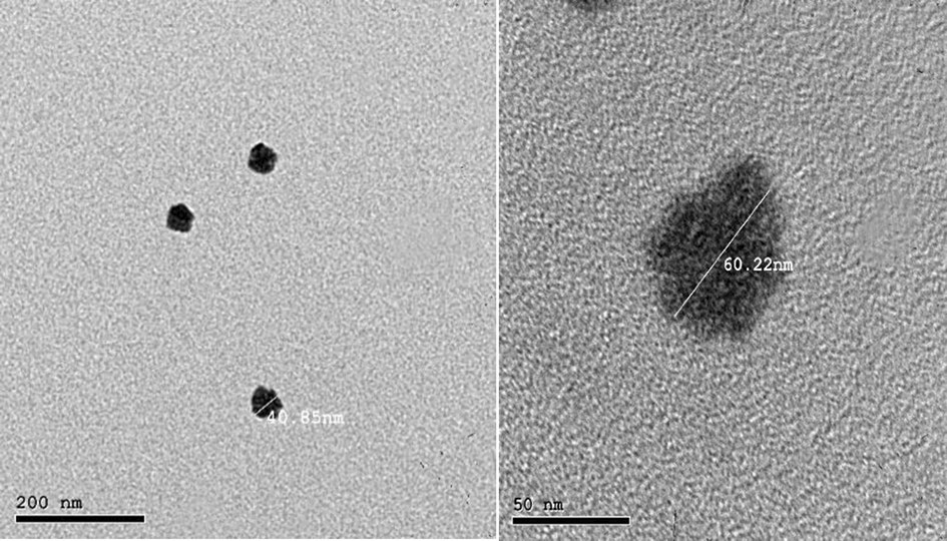
TEM images of beta-carotene nanodispersion using solvent displacement method at a volume ratio of 1:9 (water phase: organic phase) using tween 80 showing the morphology at low magnification in the left and at high magnification in the right.

TEM images revealed that the morphology of the prepared nanodispersions was nearly spherical in shape. Nano-Lut exhibited a smaller particle size, measuring 35.3 ± 7.69 nm, compared to Nano-Bc, which had a particle size of 53.6 ± 21.3 nm (Figs. 1 and 2).

### FTIR results

**Figure 3:**
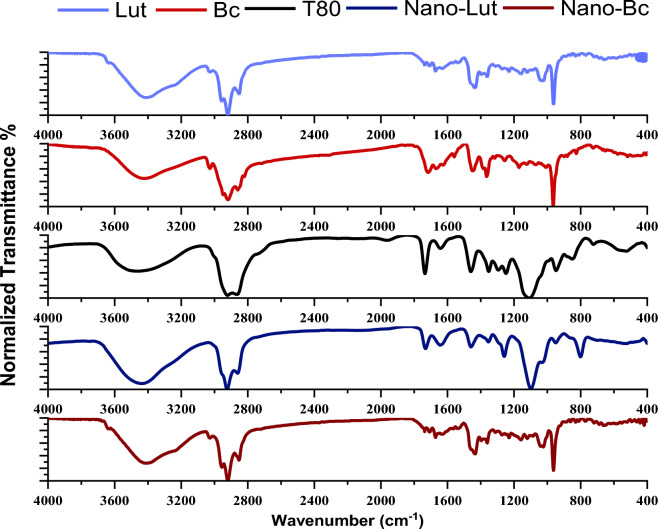
Normalized FTIR spectra of Lut, Bc, T80, Nano-Lut, and Nano-Bc samples in (4000 to 400 cm^−1^) region. The data were normalized and expressed as means values.

The presented data in Fig. 3 and Table 1^59–68^ demonstrate the normalized Fourier Transform Infrared (FTIR) spectra of lyophilized Lut, Bc, T80, Nano-Lut, and Nano-Bc in the region of 4000–400 cm^−1^. The stretching vibration of acyl chains –CH_2_ was observed in the range of 2800–3000 cm^−1^. Symmetric and antisymmetric stretching vibration peaks of –CH_2_ were observed for Lut and Bc in the regions of 2852.21, 2859.92 cm^−1^, and 2920.66, 2918.73 cm^−1^, respectively. T80 showed symmetric and antisymmetric stretching vibration peaks of –CH_2_ at 2864.74 and 2921.63 cm^−1^, respectively. After the preparation of nanodispersions, these peaks were shifted for Nano-Lut and Nano-Bc. The ester bond of Lut was detected at 736 cm^−1^, while Bc did not show any ester bond. Both T80 and its nanodispersion preparations had ester bond peaks in the same wavenumber range. In the region of 940-980 cm^−1^, Lut and Bc showed peaks at 963.27 and 964.23 cm^−1^, respectively, indicating the deformation vibration of the trans-conjugated alkene of the polyene chain. T80 and its nanodispersions showed peaks at around 947 cm^−1^, indicating the deformation vibrations of the C–H bonds. The mean wavenumber values of three replicates of each sample are summarized in Table 1.

**Table 1:** The chemical shifts observed for lutein or beta carotene before and after the preparation as nanodispersions^59–68^.

Peak assignment	Wavenumber (cm^−1^)	Lut	Bc	T80	Nano-Lut	Nano-Bc
The deformation/bending vibrations of the C–H bonds	940–950	–	–	947.84	949.77	947.84
The deformation vibration of trans-conjugated alkene in the polyene chain	950–980	963.27	964.23	–	–	–
The symmetric vibrations of the CH_3_ groups	1350–1390	1361.51	1364.39	1351.86	1354.75	1352.82
The antisymmetric vibrations deformation of CH_3_ and Scissor vibrations of CH_2_ groups	1450–1465	1450.21	1449.24	1459.85	1458.89	1460.81
Stretching vibrations of C=C	1600–1700	1671.02	1669.09	1643.05	1644.98	1642.09
Oscillations of C=O bonds	1720–1770	1736.58	–	1735.62	1731.76	1733.69
CH_2_ symmetric stretching vibration	2800–2865	2852.21	2859.92	2864.74	2860.88	2861.84
CH_2_ antisymmetric stretching vibration	2916–2926	2920.66	2918.73	2921.63	2924.52	2922.59

### Stability study

**Figure 4:**
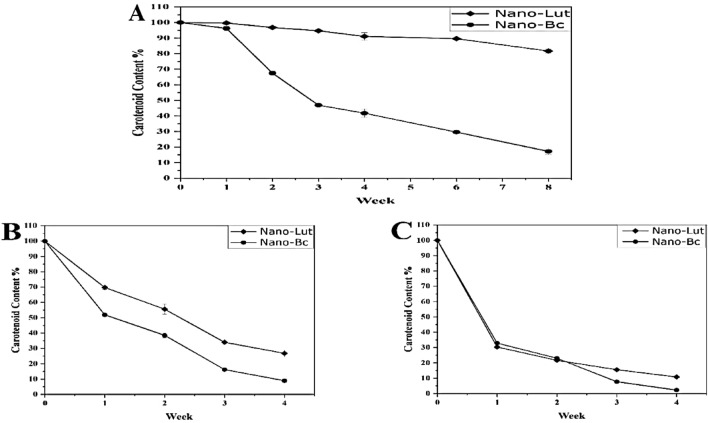
Carotenoid content associated with nanodispersion systems stored at 4 °C (**A**), 30 °C (**B**), and 40 °C (**C**) measured spectrophotometrically at 460 and 452 nm for Bc and Lut, respectively.

**Figure 5:**
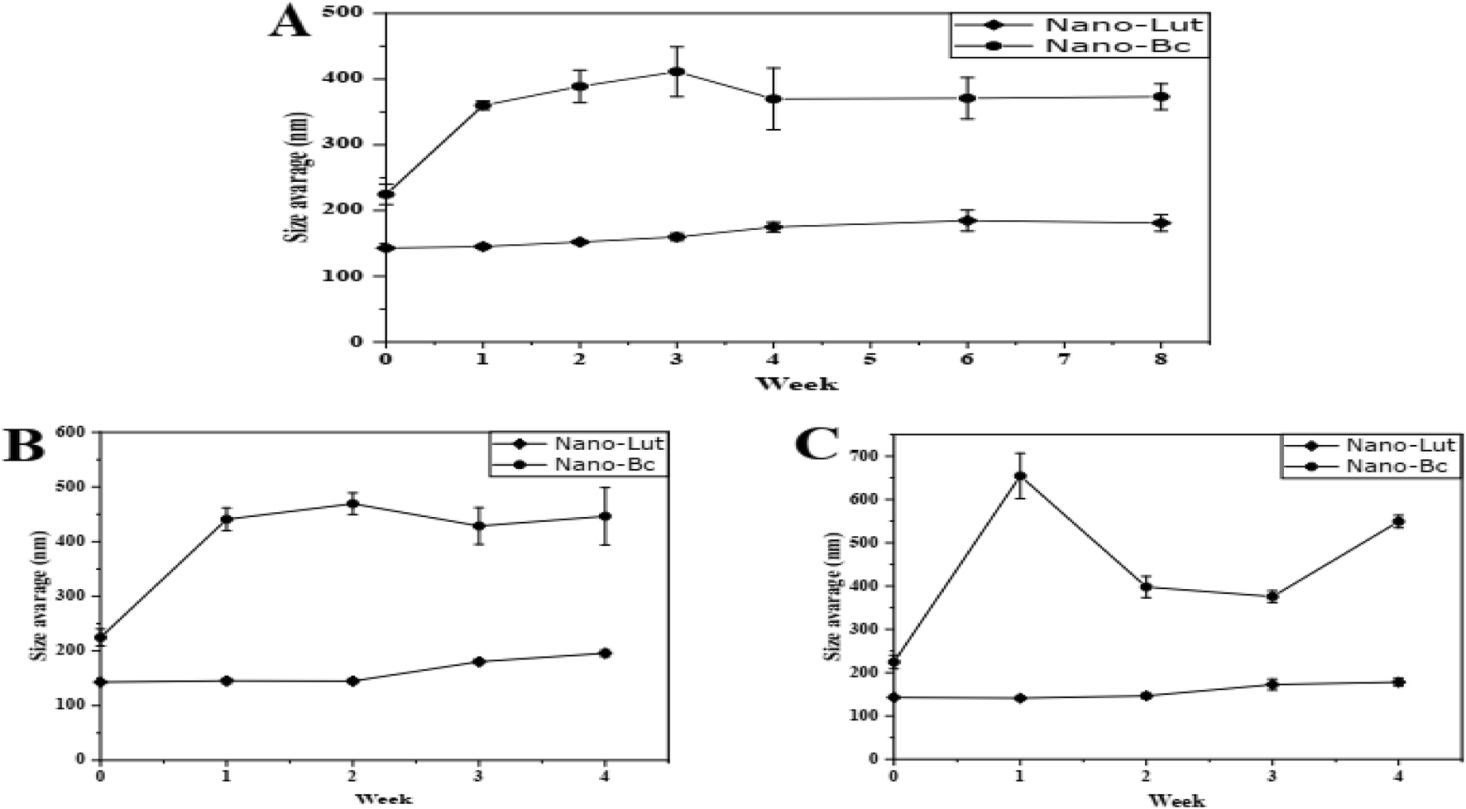
Average size associated with nanodispersion systems stored at 4 °C (**A**), 30 °C (**B**), and 40 °C (**C**), measured by using Zetasizer with a particle sizing system.

The stability of the prepared nanodispersions was examined at different storing temperatures (4, 30, and 40 °C) by comparing the drug content (Fig. 4), hydrodynamic diameter (Fig. 5), PDI, and ZP values (Tables 2 and 3) for the fresh samples and the same samples at each interval. Nano-Lut showed more stability than Nano-Bc at all tested temperatures for all parameters. Some aggregations were observed after 1 week of storing Nano-Bc at 30 and 40 °C, this aggregation was not seen with Nano-Lut at these temperatures. The best-storing temperature of the prepared nanodispersions was 4 °C and there were no aggregation or precipitations observed during the 8 weeks. Nano-Lut mean size was 142.93 ± 1.65 nm with drug content of 98.41 ± 0.94% at zero time, after 8 weeks at 4 °C the size increased to 181.10 ± 21.73 nm and drug content decreased to 79.88 ± 3% which was considered acceptable change. At 30 and 40 °C, the mean size increased insignificantly to 195.733 ± 10.45 and 177.97 ± 15.75 nm, respectively, but drug content decreased significantly to 26.386 ± 3.75 and 10.672 ± 0.794%, respectively, after 4 weeks. While Nano-Bc mean size was 224.63 ± 26.9 nm with drug content of 89.46 ± 1.28% at zero time, after 8 weeks at 4 °C the size increased significantly to 373.17 ± 34.08 nm and drug content decreased to 15.42 ± 3.34%. At 30 and 40 °C, the mean size increased significantly to 446.33 ± 90.83 and 415.7 ± 21.11 nm, respectively, and drug content decreased significantly to 8.01 ± 1.5 and 2.002 ± 0.523%, respectively, after 4 weeks.

**Table 2:** Lutein nanodispersion stability.

_Parameter_	^Interval (week)^						
	0	1	2	3	4	6	8
PDI at 4 °C	0.17 ± 0.01	0.28 ± 0.01	0.25 ± 0.03	0.31 ± 0.03	0.29 ± 0.05	0.31 ± 0.05	0.39 ± 0.02
PDI at 30 °C	0.17 ± 0.01	0.19 ± 0.01	0.18 ± 0.02	0.26 ± 0.01	0.35 ± 0.02	–	–
PDI at 40 °C	0.17 ± 0.01	0.226 ± 0.03	0.203 ± 0.03	0.298 ± 0.03	0.314 ± 0.04	–	–
Zeta potential (mV) at 4 °C	− 12.3 ± 0.1	− 13.4 ± 1.6	− 12.9 ± 1.4	− 11.1 ± 0.3	− 13.9 ± 1.3	− 15.7 ± 1.7	− 14.1 ± 1.2
Zeta potential (mV) at 30 °C	− 12.3 ± 0.1	− 13.3 ± 0.5	− 15.4 ± 0.6	− 14.2 ± 0.5	− 13.6 ± 0.7	–	–
Zeta potential (mV) at 40 °C	− 12.3 ± 0.1	− 10.26 ± 0.6	− 12.8 ± 0.4	− 11.5 ± 0.4	− 10.51 ± 0.4	–	–

**Table 3:** Beta-carotene nanodispersion stability.

_Parameter_	^Interval (week)^						
	0	1	2	3	4	6	8
PDI at 4 °C	0.43 ± 0.05	0.51 ± 0.04	0.52 ± 0.05	0.55 ± 0.21	0.62 ± 0.08	0.66 ± 0.05	0.66 ± 0.02
PDI at 30 °C	0.43 ± 0.05	0.59 ± 0.03	0.69 ± 0.06	0.71 ± 0.08	0.79 ± 0.02	–	–
PDI at 40 °C	0.43 ± 0.05	0.55 ± 0.05	0.47 ± 0.14	0.53 ± 0.04	0.76 ± 0.1	–	–
Zeta potential (mV) at 4 °C	− 18.8 ± 0.1	− 19.5 ± 1.2	− 18.5 ± 1.2	− 18.9 ± 1.6	− 18.9 ± 0.9	− 21.6 ± 0.9	− 23.3 ± 1.7
Zeta potential (mV) at 30 °C	− 18.8 ± 0.1	− 18.8 ± 1.4	− 11.3 ± 1.5	− 5.01 ± 0.5	− 4.74 ± 0.8	–	–
Zeta potential (mV) at 40 °C	− 18.8 ± 0.1	− 22.1 ± 1.1	− 10 ± 0.2	− 9.19 ± 0.4	− 8.34 ± 2.1	–	–

### Oxidative stress markers

**Figure 6:**
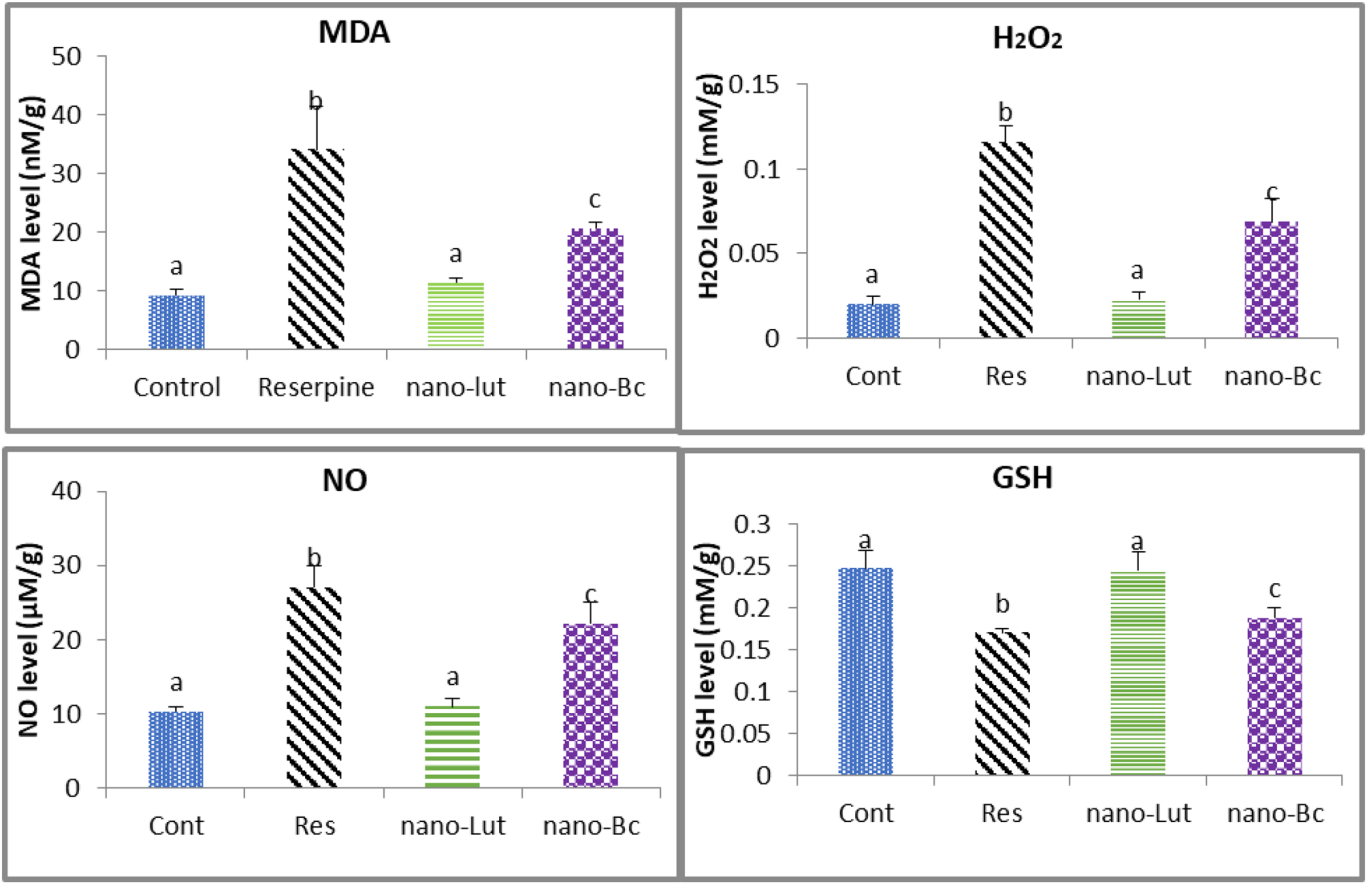
Malondialdehyde (MDA), hydrogen peroxide (H_2_O_2_), nitric oxide (NO), and reduced glutathione (GSH) concentrations in the cortical tissue of control (Cont), reserpine (Res), nano-Lutein (nano-Lut), and nano-beta-carotene (nano-Bc) treated groups of animals. Data represents the average values with the standard deviation. Bars labeled with the same letter denote non-significant differences and those with different letters denote significant differences at *P* < 0.05.

In this study, the levels of MDA, H_2_O_2_, NO, and GSH in the cortical brain tissue were assessed for all animal groups. Subcutaneous injections of reserpine (1 mg/kg) resulted in a significant increase of MDA by 266.7% from 9.661 ± 2.140 to 33.545 ± 10.514 nM/g, H_2_O_2_ by 30.6% from 0.023 ± 0.011 to 0.116 ± 0.023 mM/g, and NO by 12.3% from 9.323 ± 1.582 to 27.117 ± 6.909 µM/g, while GSH decreased by 74% from 0.247 ± 0.058 to 0.171 ± 0.013 mM/g. However, a dose of 0.5 mg/kg Nano-Lut was found to be more effective in its antioxidant action than Nano-Bc, as shown by the results of the oxidative stress parameters. Specifically, GSH levels returned to their control values, which agreed with the cell-free-based antioxidant activity assays in our previous results^48^. In contrast, treatment with the same dose of Nano-Bc did not restore the measured parameters to their control values (Fig 6). In the case of Nano-Bc, MDA, H_2_O_2_, NO, and GSH were 25.736 ± 4.83 nM/g, 0.069 ± 0.031 mM/g, 22.224 ± 6.781 µM/g, 0.192 ± 0.03 mM/g, respectively.

### Monoamine levels in cortical tissue

**Figure 7:**
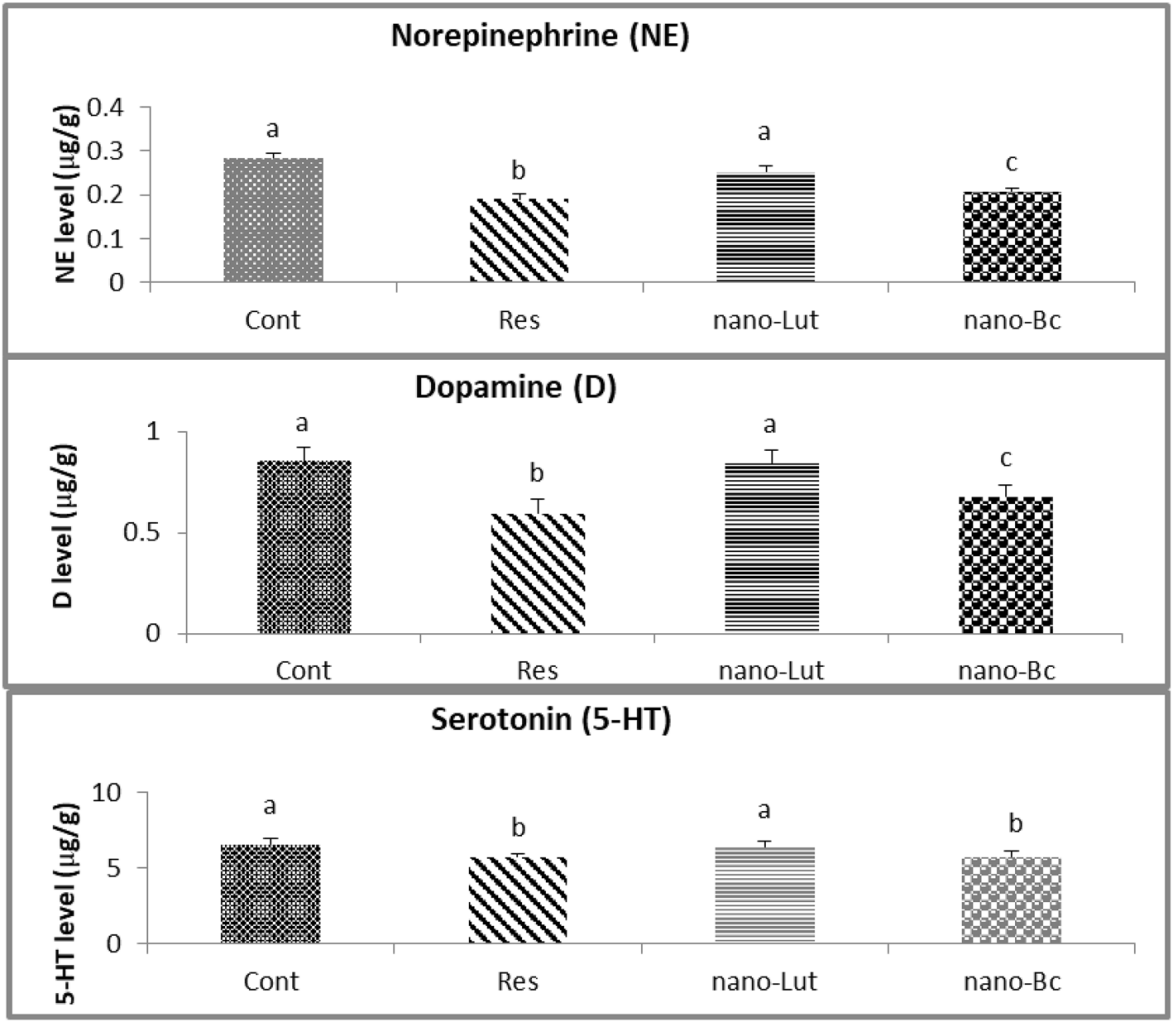
Norepinephrine (NE), dopamine (D), and serotonin (5-HT) levels in the cortical tissue of control (Cont), reserpine (Res), nano-Lutein (nano-Lut), and nano-beta-carotene (nano-Bc) treated groups. Bars represent the average values with the standard deviation. Bars labeled with the same letter denote non-significant differences and those with different letters denote significant differences at *P* < 0.05.

To assess the levels of NE, DA, and 5-HT neurotransmitters in different animal groups, fluorescence measurements of monoamines were performed on cortical brain tissue. Results showed that the cortex of rats treated with reserpine had reduced levels of NE by 35.7% from 0.303 ± 0.047 to 0.199 ± 0.043 µg/g, DA by 30.6% from 0.856 ± 0.175 to 0.595 ± 0.163 µg/g, and 5-HT by 12.3% from 6.596 ± 1.017 µg/g to 5.736 ± 0.646 µg/g. However, treatment with Nano-Bc or Nano-Lut mitigated or restored the decrease in monoamine concentration to a great extent. In the case of Nano-Bc, levels of NE, DA, and 5-HT were 0.252 ± 0.055 µg/g, 0.677 ± 0.137 µg/g, and 5.766±0.989 µg/g, respectively. In the case of Nano-Lut, levels of NE, DA, and 5-HT were 0.252 ± 0.037 µg/g, 0.845 ± 0.149 µg/g, and 6.467 ± 1.061 µg/g, respectively. Specifically, Nano-Lut was able to recover the three neurotransmitters to control values, while Nano-Bc failed to reach the same level of recovery **(**Fig 7**)**.

### ECoG analysis

**Figure 8:**
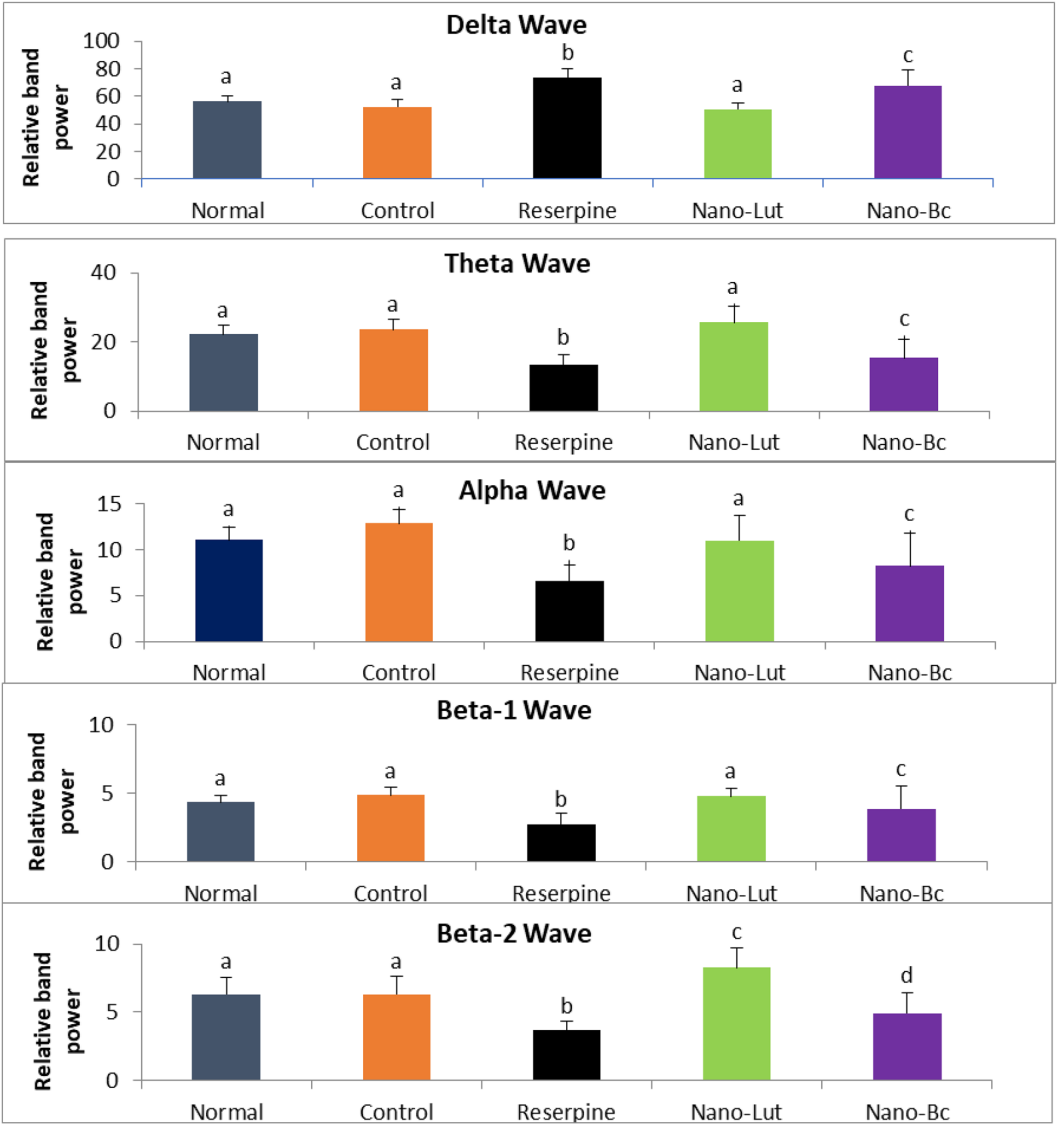
ECoG relative band power of delta (1–4 Hz), Theta (4.1–7 Hz), alpha (8–13 Hz), beta-1 (13.1–20 Hz), and beta-2 (20.1–35 Hz) in normal, control, reserpine, Nano-lutein, and Nano-beta-carotene groups of animals. Bars represent the relative band power with error bars representing the standard deviation. The same letters above the bars represent non-significant differences, while different letters represent significant differences at *P* < 0.05.

Different groups of rats had their cortex ECoG signals recorded. Quantitative spectral analysis was performed on the recorded signals. As shown in Fig. 8, there was no significant difference in delta wave between normal (no injection) and control (drug vehicle injection), with a relative band power of 56.177 ± 5.89 and 52.42 ± 7.195, respectively. After three days of reserpine administration, there was a significant increase in delta wave (40.5%) with relative band power of 73.68 and a significant decrease in other frequency bands. The change of theta wave was insignificant difference between normal (with band power of 22.117 ± 3.956) and control (with band power of 23.573 ± 4.405) and decreased to 13.371 ± 4.041 after reserpine administration. The same pattern was observed at alpha, beta-1, and beta-2 band, the normal group had relative band powers of 11.067 ± 1.98, 4.336 ± 0.767, and 6.302 ± 1.743, respectively. Control group was 12.829 ± 2.14, 4.853 ± 0.836, and 6.326 ± 1.874 of alpha, beta-1, and beta-2 band, respectively, while after reserpine was 6.562 ± 2.456, 2.733 ± 1.167, and 3.652 ± 0.9732 of alpha, beta-1, and beta-2 band, respectively.

**Figure 9:**
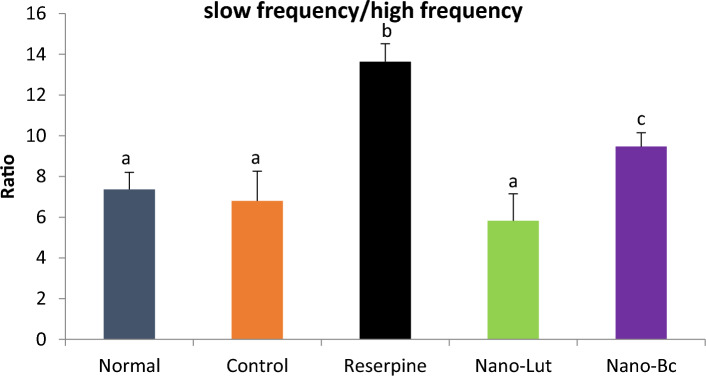
Slow frequencies (delta & theta) fast frequencies (beta-1 & beta-2) ratios in normal, control, reserpine, nano-lutein, and nano-beta-carotene groups of animals. Data represent the ratio values and error bars represent the standard deviation. Data labeled with the same letter denote non-significant difference and with different letters denote significant difference at *P* < 0.05.

However, treating these animals for two weeks with 0.5 mg/kg Nano-Lut resulted in the restoration of the normal frequency band distribution and a return to normal and control-like values of these electrical brain waves. Nano-Lut showed relative band power of 50.408 ± 7.025, 25.560 ± 6.762, 10.988 ± 3.915, 4.774 ± 0.789, and 8.2694 ± 2.009 of delta, theta, alpha, beta-1, beta-2 band, respectively. The same dose of Nano-Bc treatment did not achieve the same level of normal distribution recovery as Nano-Lut treatment. Nano-Bc showed relative band power of 67.643 ± 15.88, 15.364 ± 7.729, 5.225 ± 3.503, 3.847 ± 2.32, and 4.92 ± 2.178 of delta, theta, alpha, beta-1, beta-2 band, respectively. The ratio of slow waves (delta and theta) to fast waves (beta-1 and beta-2) demonstrates the recovery action of Nano-Lut and Nano-Bc on brain waves following the significant increase obtained following reserpine injection (Fig. 9).

### Pain threshold results

The results of measuring pain response latency in all animal groups are presented in Table 4. It was found that reserpine reduced pain response latency by 32.2% compared to the control value. However, treatment with Nano-Bc increased this latency by 28.1% compared to the control. On the other hand, Nano-Lut treatment was able to achieve a negligible (insignificant) difference in latency, which was only 1.9% lower than the control value.

**Table 4:** Pain threshold in the control, reserpine (Res), lutein nanodispersion (Nano-Lut) treated, and beta carotene nanodispersion (Nano-Bc) treated groups.

Group	Pain response latency (min)
Control	6.1 ± 0.4
Res	4.1 ± 0.2*
Res + Nano-Lut	5.99 ± 0.26
Res + Nano-Bc	4.39 ± 0.29*

Data represents average values ± standard deviation values. *Denotes significant difference from control at *P* < 0.05.

### Open field results

Locomotor activity was tested in all animal groups, and the findings are provided in Table 5. In reserpinized animals, the number of squares crossed, grooming, raising, sniffing, and mobile time were considerably reduced, while the central immobility time was significantly enhanced compared to the control group. After Nano-Lut treatment, all decreasing assessments improved, particularly the time spent outside of the central zone. Nano-Bc, on the other hand, failed to recover the open field parameters to the level reached by Nano-Lut.

**Table 5:** Parameters of OFT in the control, reserpine (Res), lutein nanodispersion (Nano-Lut) treated, and beta carotene nanodispersion (Nano-Bc) treated groups.

	Square crossing	Groom	Rearing	Sniffing	Mobile		Immobile	
					Center	Outer	Center	Outer
Control	200 ± 26	4.23 ± 0.4	34 ± 3.5	53.2 ± 4.7	16 ± 1.8	231 ± 26.1	47 ± 7.8	311 ± 26.1
Res	0.91 ± 0.3	1.6 ± 0.3	0	18.2 ± 1.2	587.5 ± 4.8	0	12.5 ± 0.8	0
Res + Nano-Lut	90.3 ± 4.5	5 ± 0.6	5.5 ± 0.6	35.8 ± 2.7	5.17 ± 1.1	244.3 ± 28.1	21.83 ± 7.85	328.7 ± 31.9
Res + Nano-Bc	19.1 ± 4.3	2.83 ± 0.5	3.5 ± 0.4	9.8 ± 0.8	5.5 ± 0.99	451.2 ± 24.8	3.67 ± 0.67	193.2 ± 24.6

Data represent average values ± standard deviation.

## Discussion

Carotenoids are a diverse group of natural pigments that are found in many fruits and vegetables^70^. They are well-known for their antioxidant and anti-inflammatory properties, which have been extensively studied considering their potential to prevent chronic diseases such as cancer, cardiovascular disease, and age-related macular degeneration^71,72^. While the physical health benefits of carotenoids are well-established, their potential role in promoting mental health and neurological function has received less attention. In the present study, two important carotenoids, lutein, and beta-carotene, have been evaluated for their potential to promote antioxidation, antidepressant effects, and anti-nociception properties for the treatment of FM induced in animals.

**Figure 10:**
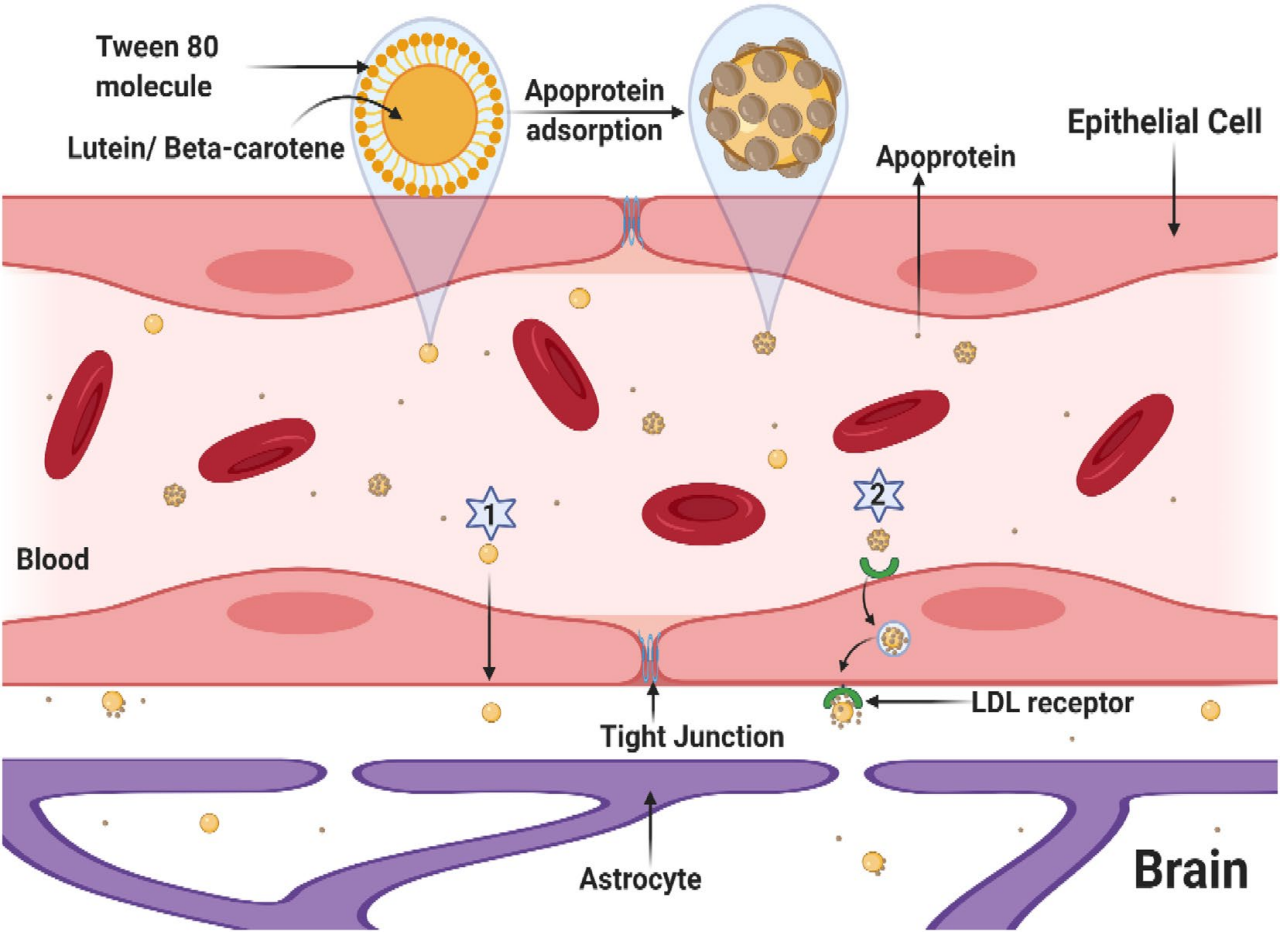
The drawing shows the enhanced brain uptake of Nano-Bc and Nano-Lut by apoprotein adsorption on the nanoparticle surface via receptor-mediated endocytosis (binding to the BBB low-density lipoprotein receptors) (1) and simple diffusion (2) then the carotenoid released from nanoparticles upon endocytosis and transcytosis through the BBB endothelial cells.

Hydrophobicity, or the inability of a molecule to dissolve in water, can be a challenge in the use of carotenoids within living organisms. To address this issue, various methods have been developed to enhance the solubility and bioavailability of carotenoids. For instance, emulsions and micelles can encapsulate carotenoids, improving their solubility in water-based solutions^48^. In the present study, nanodispersion was used as a technique to enhance the solubility and bioavailability of carotenoids. This method helped to overcome the hydrophobicity of carotenoids and produce particles that are small enough to be easily absorbed by the body. The nanodispersion system was selected because of the easy formulation, cost-efficiency, and ability to scale up. Moreover, nanodispersion has been shown to facilitate the crossing of the blood–brain barrier (BBB)^44,47^ as summarized in Fig. 10.

TEM showed a more homogenous shape and size for Nano-Lut compared with Nano-Bc which agreed with the DLS results. The –OH group of lutein may play a role in controlling the orientation and homogenizing the distribution of lutein molecules in the nanodispersion which is not in beta-carotene molecules.

FTIR analysis indicated the active ingredient interacts with the nanoparticle carrier composition. Lut and Bc showed characteristic peaks of 963.27 and 964.23 cm^−1^, receptively, which represent the deformation vibration of trans-conjugated alkene in the polyene chain^63,66,67^, this peak disappeared or may be overlapped with the appearance of the C-H deformation vibration band of T80 after formulated as nanodispersion at 947.84, 949.77, and 947.84 of T80, Nano-Lut, and Nano-Bc, respectively. Peaks in the range of 1350–1390 and 1450–1465 cm^−1^ represent the symmetric and antisymmetric vibrations of the CH_3_ group^63–66^. These peaks revealed shifting after nanodispersion formulation from 1361.51 to 1354.75 cm^−1^ and 1450.21 to 1458.89 cm^−1^ with lutein and from 1364.39 to 1352.82 cm^−1^ and 1449.24 cm to 1460.81 cm^−1^ where T80 showed peaks at 1351.86 cm^−1^ and 1459.85 cm^−1^. Peaks in the range of 2800–2865 cm^−1^ and 2916–2926 cm^−1^ represent the symmetric and antisymmetric vibrations of the CH_2_ group^60–69^. These peaks revealed also shifting after nanodispersion formulation from 2852.201 to 2860.88 cm^−1^ and 2920.66 to 2924.52 cm^−1^ with lutein and from 2859.92 to 2861.84 cm^−1^ and 2918.73 to 2922.59 cm^−1^ where T80 showed peaks at 2864.74 cm^−1^ and 2921.63 cm^−1^. The same happened in the case of C=C stretching vibration, as the peaks of Nano-Lut and Nano-Bc shifted to the T80 range. Oscillations of the C=O bond peak appeared in Nano-Bc and there were shifts for this peak in all samples. All FTIR results indicate the successful formulation of lutein and beta-carotene as nanodispersions.

Gravitational separation, aggregation (coalescence), and Ostwald ripening are the most well-known causes of nanodispersion instability^73^. As expected Nano-Lut was more stable than Nano-Bc as lutein molecules are more polar than beta-carotene^74^ so lutein molecules can stand in the nanodispersion system containing water and T80 with hydrogen bonds between oxygen and hydrogen atoms of lutein, T80, and water beside the main interactions between the lutein polyene chain and T80 acyl chains, where in case of beta-carotene, the interaction based on the polyene chain and acyl chain only. The lutein content of Nano-Lut decreased only by 18% after 8 weeks of storing at 4 °C but the beta-carotene content of Nano-Bc decreased by 73%. The carotenoid content ratio of Nano-Bc after storing at 4 °C for 1 week was insignificant compared with Nano-Lut but the size and PDI differed significantly. However, the zeta potential of Nano-Bc was higher than Nano-Lut, and zeta potential values did not change significantly with the significant difference in carotenoid content, size, and PDI. It should be noted that zeta potential is one of the stability factors not the only indicator. Nano-Lut kept its characterizations after storing at 30 °C more than Nano-Bc but this did not happen after storing at 40 °C. These stability results can strongly guide the workflow with Nano-Lut and Nano-Bc. Increasing the size of Nano-Bc than Nano-Lut and less negative zeta potential may be additional reasons for Nano-Lut giving better results as a treatment for our animal model.

Oxidative stress and abnormalities in monoamine neurotransmitters have been implicated in the pathogenesis of FM^14–16^. ROS can damage cells and tissues and contribute to the development of chronic pain syndromes such as FM^12,13^. Studies have shown that patients with FM have higher levels of oxidative stress markers, such as MDA and protein carbonyls, compared to healthy individuals^75^. In addition, antioxidant defenses, such as glutathione and superoxide dismutase, are often reduced in FM patients, further exacerbating oxidative stress^76^. Additionally, abnormalities in monoamine neurotransmitters, such as 5-HT, NE, and D, have also been implicated in the pathogenesis of FM^19–21^. These neurotransmitters play important roles in regulating pain perception, mood, and stress responses. Several studies have shown that FM patients have lower levels of monoamine neurotransmitters, which may contribute to the development of chronic pain and depressive symptoms^20,21^.

In the present study, successive reserpine (1 mg/kg, SC) administration resulted in a significant increase in oxidative stress markers MDA, H_2_O_2_, and NO accompanied by a significant decrease in GSH^31,32^. Reserpine as a monoamine-depleting agent that blocks the transport of these neurotransmitters into their vesicles by inhibiting the activity of vesicular monoamine transporter (VMAT) can contribute to an increase in oxidative stress in nerve cells and exhaustion of antioxidant molecules such as GSH. The blocking of neurotransmitters' transportation to their vesicles increases their concentration in the cytosol and this in turn exposes them to oxidative deamination by monoamine oxidase (MAO), the enzyme found in the mitochondrial outer membrane. This reaction led to the production of H_2_O_2_ as a by-product and enhanced the existence of free radicals in the cell. The free radicals attack the phospholipids and cause their peroxidation and the antioxidant activity of the cell is drained in mitigating these reactions^33,77,78^.

On the other hand, the reduction of monoamine neurotransmitters availability caused by reserpine can lead to negative side effects associated with depression, pain signal processing abnormality, cognition impairment, and motor activity alteration^332–34^. Therefore, the changes observed in the pain threshold test, OFT, and their reflection on brain electrical activity (EEG) are suggested to be a direct consequence of monoamine depletion and oxidative stress induced by reserpine^52^.

Lutein and beta-carotene have a treatment potential due to their antioxidant activity. Both act as antioxidants by neutralizing free radicals and reducing oxidative stress in cells. They can scavenge free radicals and prevent them from causing cellular damage. They are also able to modulate the levels of monoamine neurotransmitters and restore them to their normal levels in the brain^52,79,80^. In the present study, administration of Nano-Lut or Nano-Bc for 14 days has a positive effect on the brain cortical tissue oxidative stress and monoamine levels. Interestingly, nano-Lut proved to be more effective in improving antioxidant status and restoring monoamine levels. This greater effectiveness of Nano-Lut needs to be further elucidated, however, it might be attributed to its ability to cross the BBB more easily than Nano-Bc and its stability in addition to Lut and Nano-Lut proved in vitro antioxidant potency more than Bc and Nano-Bc in our previous work^48^.

Furthermore, the treatment capacity of Nano-Lut and Nano-Bc obtained in the present study could be associated with the reported impact of carotenoids as MAO inhibitors. Inhibition of the activity of MAO by carotenoids leads to the elevation of the level of the monoamine and the reduction of oxidative stress load on the cells^81^. Despite the exact mechanism by which carotenoids inhibit the activity of MAO is not fully elucidated, some studies reported results that emphasize this effect^82^. Recent investigations have pointed out that the antioxidant properties of natural compounds such as carotenoids may be associated with the inhibition of MAO activity. Other studies refer to modulation of the gene expression which is involved in the regulation of MAO activity by carotenoids.

The ECoG, a recording of the electrical activity of the surface of the brain, can provide insights into brain function and activity. Additionally, it may reflect the positive and negative alterations in the brain neurotransmitters. Therefore, ECoG is considered a direct measure that can be used to monitor the nervous system activity in health and disease status^52^. In the present study, reserpine administration led to a significant increase in delta wave power, and a decrease in theta, alpha, and beta wave powers. On the other hand, Nano-Lut and to some extent, Nano-Bc succeeded in re-normalization of most of the brain wave changes induced by reserpine. Studies have shown that changes in EEG patterns are associated with changes in cognitive function, vigilance, and arousal in rats. Increased theta and beta wave activity in the cortex has been associated with improved attention and memory performance in rats. Delta, alpha, and beta waves have also been linked to cognitive function and arousal in rats. For example, increased delta activity in the cortex has been associated with a decrease in arousal, and a decrease in theta activity is associated with attenuation in motor activity^83,84^. Therefore, the ECoG results obtained in the present study emphasize the adverse effect of reserpine on the brain and the treatment potential of Nano-Lut in restoring these activities.

Parallel to these animal results, studies have shown that there are differences in the EEG patterns of patients with FM compared to healthy individuals. These differences suggest that FM patients may have alterations in brain function and activity that contribute to the development of FM symptoms^85,86^. A study found that FM patients had increased alpha and theta wave activity in the frontal cortex, which is associated with decreased arousal and attention. This suggests that FM patients may have difficulty staying alert and focused, which could contribute to FM-related fatigue and cognitive impairment. Another study found that FM patients had decreased delta and beta wave activity in the parietal cortex, which is involved in sensory processing and spatial awareness^87,88^. This suggests that FM patients may have altered sensory processing and perception, which could contribute to FM-related pain and sensitivity. Other studies have reported alterations in high-frequency activity in FM patients, which is associated with attention, memory, and cognitive processing and may be involved in executive function and decision-making^89,90^.

Pain threshold tests and OFT are commonly used to assess animal behavior related to FM^91^. Pain threshold tests measure an animal's sensitivity to pain, and increased sensitivity to pain, or hyperalgesia, is often observed in animal models of FM. OFT assesses an animal's exploratory and anxiety-like behaviors, and animals with FM-like symptoms often exhibit decreased exploratory behavior and increased anxiety-like behavior in these tests. The present study demonstrated that Nano-carotenoids have the potential to alleviate FM-related symptoms induced by reserpine in animal models, as evidenced by changes in behavioral outcomes. Specifically, treatment with Nano-Lut restored pain threshold latency and significantly increased square crossings and exploratory behavior in the OFT, indicating recovery of animal behavior after treatment. However, the effects of nano-Bc on the behavioral outcomes were less pronounced and may require further investigation to draw solid conclusions about its efficacy.

In a study conducted by Oliveira et al.^92^, α-Terpineol and α-Terpineol-complex-β-cyclodextrin were produced as potential oral analgesics for an acid-saline animal model of FM in male mice. The mice were administered doses of 25, 50, or 100 mg/kg once daily for 10 days. The researchers found that the addition of β-cyclodextrin enhanced the efficacy of α-Terpineol alone, leading to a decrease in mechanical hyperalgesia across all dosage levels.

Another investigation focused on oral melatonin as a treatment for FM induced in rats using reserpine. Daily doses of melatonin at 2.5 and 5 mg/kg were administered for a period of 2 months. The results showed improvements in motor activity, musculoskeletal morphology, and a decrease in inflammation and oxidative stress^93^. In a similar approach, melatonin was studied in combination with folic acid. Rats induced with reserpine were treated with a daily dose of 10 mg/kg melatonin plus 3 mg/kg folic acid for 21 days. This combination treatment significantly improved behavior, reduced mast cell infiltration and decreased oxidative and nitrosative stress and inflammation compared to reserpine-treated rats. The combined administration of melatonin and folic acid demonstrated higher efficacy than when administered separately^92^.

The natural polyphenol flavonoid fisetin was also investigated as a potential treatment for FM. Rats induced with FM using the reserpine animal model were administered different doses of fisetin (10 and 25 mg/kg) once a week for 3 weeks. The results showed a decrease in immobility time, increased withdrawal latency and paw threshold, and normalization of monoamine and monoamine derivative levels^94^.

Furthermore, in our previous study^52^, the reserpine-induced FM animal model was treated with lutein or beta-carotene encapsulated in cationic liposomes, with a daily intravenous dose of 0.25 mg/kg for 14 days. These formulations, particularly the lutein formula, significantly regulated monoamines, oxidative and nitrosative stress, mobility, and pain threshold.

The reserpine animal model is widely recognized as the most commonly employed animal model for FM due to its numerous validities for human disease compared to other animal models^34,35^. However, Further investigation of the other models could provide more evidence of the efficacy of the present therapeutic formulations.

In conclusion, the present study provides compelling evidence, supported by the obtained results, for the efficacy of nanodispersions of lutein and beta-carotene in treating FM-related symptoms induced by reserpine in animal models. The dual action of the nano-carotenoids, as both antioxidant and antidepressant agents, is believed to account for their beneficial effects in treating FM. This study highlights their potential as a treatment option across various levels, including biochemical, bioelectrical, and behavioral. However, further research is needed to fully elucidate the mechanisms by which nano-carotenoids exert their effects on the nervous system.

## Data Availability

The datasets used and/or analyzed during the current study are available from the corresponding author upon reasonable request.
